# Identification of predictive biomarkers and dose optimization for camrelizumab combined with apatinib in the treatment of advanced hepatocellular carcinoma: a quantitative systems pharmacology approach

**DOI:** 10.3389/fimmu.2026.1617227

**Published:** 2026-02-17

**Authors:** Weikun Huang, Guihui Tu, Dandan Li, Chenyu Wang, Jianxing Zhou, Zheng Jiao, Lin Yang

**Affiliations:** 1 Department of Pharmacy, Clinical Oncology School of Fujian Medical University, Fujian Cancer Hospital, NHC Key Laboratory of Cancer Metabolism, Fuzhou, Fujian, China; 2 Pharmacy Department, Chongqing Emergency Medical Center, Chongqing University Central Hospital, Medical College, Chongqing University, Chongqing, China; 3 Shanghai Chest Hospital, School of Medicine, Shanghai Jiao Tong University, Shanghai, China; 4 Department of Pharmacy, Fujian Medical University Union Hospital, Fuzhou, Fujian, China; 5 Department of Pharmacy, Shanghai Chest Hospital, Shanghai Jiao Tong University School of Medicine, Shanghai, China

**Keywords:** advanced hepatocellular carcinoma, apatinib, biomarkers, camrelizumab, dose optimization, quantitative systems pharmacology

## Abstract

**Introduction:**

The combination of camrelizumab and apatinib represents a promising treatment strategy for patients with advanced hepatocellular carcinoma (aHCC). However, the specific patient populations that may benefit from this combination therapy, as well as the changes in efficacy after adjusting the medication regimen to avoid serious adverse reactions, remain uncertain.

**Methods:**

We employ a quantitative systems pharmacology (QSP) approach to address these significant clinical issues. A QSP model is established by integrating pharmacokinetic data of camrelizumab and apatinib, generating a virtual patient cohort for rapid and reliable virtual clinical studies.

**Results:**

Ultimately, our model identifies the pre-treatment CD8+/Treg ratio, CD4+/Treg ratio, and the density of myeloid-derived suppressor cells (MDSCs) as key predictive biomarkers. Furthermore, through computer-simulated clinical trials, we find that reducing the dose of apatinib in combination therapy to 125 mg can still achieve therapeutic effects comparable to the original dose.

**Discussion:**

These findings provide valuable insights for future drug development and clinical trial design.

## 1 Introduction

Hepatocellular carcinoma (HCC) is the most prevalent form of liver cancer, with both incidence and mortality rates steadily rising worldwide (1). Chronic infections with hepatitis B virus (HBV) and hepatitis C virus (HCV) are the primary risk factors contributing to the development of HCC (2). Despite significant advancements in disease screening technologies, the majority of HCC patients are diagnosed at an advanced stage, referred to as advanced hepatocellular carcinoma (aHCC) (3). At this advanced stage, traditional therapeutic approaches—including liver transplantation, surgical resection, and conventional chemotherapy—exhibit limited efficacy, thereby rendering systemic therapy the primary treatment option for patients with aHCC (4).

Systemic treatment for aHCC has long posed a significant therapeutic challenge. Prior to 2017, treatment predominantly relied on anti-angiogenic tyrosine kinase inhibitors (TKIs) such as sorafenib (5). However, the approval of novel first- and second-line therapies has introduced immune checkpoint inhibitor (ICI)-based combination strategies, which have reinvigorated treatment possibilities for aHCC, thereby expanding the range of available therapeutic options (6, 7). Clinical trials, including RESCUE and CARES-310, have demonstrated that the combination of camrelizumab (an ICI) and apatinib (a TKI) significantly prolongs overall survival (OS) and progression-free survival (PFS) in patients with aHCC (8, 9). In light of these promising findings, the Chinese Society of Clinical Oncology (CSCO) Hepatocellular Carcinoma Expert Committee has recommended the camrelizumab-apatinib combination as one of the first-line treatment options for aHCC in its clinical guidelines (10).

Despite the significant efficacy of this combination therapy, the objective response rates (ORR) in the first-line treatment arms of the RESCUE and CARES-310 trials were 34.3% and 25%, respectively. This indicates that a substantial proportion of patients with aHCC do not respond optimally to the camrelizumab-apatinib combination. Therefore, identifying predictive biomarkers to better select patients likely to benefit from this combination therapy has become crucial. The current study investigates a limited range of biomarkers with a relatively small sample size, which affects the reliability of the selection results (11, 12). Additionally, a high proportion of patients required dose adjustments or changes in treatment frequency due to adverse reactions. In the RESCUE and CARES-310 trials, 60.6% (20 patients) and 72.1% (137 patients) of patients experienced grade 3 or higher adverse drug reactions, leading to treatment interruptions or dose modifications. However, the impact of dose reductions on treatment efficacy remains inadequately explored. Given these limitations, exploring reliable biomarkers and optimizing treatment strategies in larger cohorts has become urgent.

Virtual clinical trials, which enable rapid, cost-effective, and efficient drug evaluation in large populations, appear to be a promising solution (13). Quantitative systems pharmacology (QSP), leveraging demographic data, disease mechanisms, and drug properties, has emerged as a preferred approach for conducting virtual clinical trials (14). According to the International Society of Pharmacology, QSP is predominantly applied in the development of cancer immunotherapies to address critical issues such as dose and schedule optimization, combination therapy evaluation, biomarker discovery, and clinical trial design (15). In 2020, Sové et al. developed a QSP platform for immuno-oncology (QSP-IO) that incorporates detailed mechanisms of key immune interactions (16). This platform facilitates the creation of QSP models of varying complexity for IO research. Utilizing this platform, researchers have developed QSP models that account for anti-cancer drug characteristics, tumor immune microenvironment dynamics, and spatial multi-omics data, thereby identifying efficacy-related biomarkers and optimizing therapeutic strategies for diseases such as breast cancer, lung cancer, and liver cancer, which contribute to the standardization of clinical drug use (17–19).

This study addresses two major challenges in the combination therapy of camrelizumab and apatinib for patients with aHCC: the relatively low ORR and the high incidence and severity of adverse reactions. To overcome these therapeutic limitations, we enhanced an existing QSP model of aHCC based on the QSP-IO platform developed by Sové et al., enabling simulation of the therapeutic effects of camrelizumab and apatinib under various dosing regimens. Our primary objective is to identify predictive biomarkers that can guide patient selection and maximize treatment benefit. Additionally, we aim to evaluate the impact of dose reduction on therapeutic efficacy and to optimize dosing strategies for improved clinical outcomes.

## 2 Materials and methods

In this study, we further refine a QSP model for aHCC based on the QSP-IO platform developed by Sové et al. to simulate the therapeutic effects of camrelizumab and apatinib. Our objective is to identify predictive biomarkers that can guide precise patient selection and ultimately enhance treatment success rates. Additionally, we aim to investigate the impact of dose reduction on treatment efficacy and optimize the dosing regimen for improved clinical outcomes.

### 2.1 Model development

The QSP model was developed using QSP-IO, a modular platform based on MATLAB (MathWorks, version R2023b, Natick, MA, USA) SimBiology, specifically designed for creating QSP models in IO research (16). The model comprises four compartments that represent different biological environments: the tumor, tumor-draining lymph nodes (LNs), central (blood), and peripheral (all other organs) compartments. This structure aims to comprehensively capture the complete virtual patient. As a modular framework, the model incorporates ten distinct modules that describe various biological components and processes. These include cancer cells, effector T cells (Teff), regulatory T cells (Treg), antigen-presenting cells (APCs), tumor-specific neoantigens, tumor-associated self-antigens, immune checkpoints, bone marrow-derived suppressor cells (MDSCs), macrophages, and therapeutic drug pharmacokinetics.

The model workflow consists of five key steps: (1) Initializing Parameters: The load_parameters function loads the initial set of model parameters; (2) Model Initialization: The simbio_init function creates a simulated biological object, integrating modules that define species, events, and rules; (3) Baseline Simulation: Initial conditions are simulated using baseline parameters and the expected initial tumor diameter; (4) Defining the Dosing Regimen: The regimen is specified, including drug dosage, dosing interval, and treatment duration; (5) Conducting Virtual Clinical Trials: Multiple parameter sets are generated to represent virtual patients, enabling simulations and analyses of clinical outcomes (20). Global uncertainty and sensitivity analyses were conducted using Latin Hypercube Sampling (LHS) and the Partial Rank Correlation Coefficient (PRCC) methods to evaluate the impact of various parameters on the observed outcomes of the model (21). In the PRCC analysis, outcome indicators such as tumor volume, Treg cell density, and macrophage density are particularly noteworthy. Parameters that exhibited significant correlations with the model outputs (PRCC values greater than 0.05 or less than -0.05, with statistical significance) were identified and further analyzed (22).

### 2.2 Model evaluation

The efficacy of the virtual patient model was assessed based on the Response Evaluation Criteria in Solid Tumors (RECIST) v.1.1 (23). RECIST v.1.1 is a classification system utilized to evaluate the response of solid tumors to treatment, primarily based on the percentage change in tumor diameter relative to the baseline or minimum diameter. Tumor diameter is defined as the sum of the longest diameters of all measurable lesions. In this study, tumors were assumed to be spherical, and tumor diameter was calculated from tumor volume.

The ORR and median duration of response (DOR) predicted by the model were calculated, with the median and 95% confidence intervals (CIs) reported. These results were subsequently compared to those from the CARES-310 clinical trial, facilitating the refinement and adjustment of the model’s parameter distributions (9). To align with the clinical setup of the CARES-310 study, the treatment duration was established at 700 days, with tumor volume assessed every 8 weeks. In the CARES-310 trial, the combination therapy of camrelizumab and apatinib yielded an ORR of 25.4% (95% CI: 20.3-31.0%) and a median DOR of 14.8 months (95% CI: 8.4-NR). Considering the significant differences in tumor progression and body tolerance between patients receiving first-line therapy and those receiving second-line therapy or higher, only the ORR from the clinical study involving non-first-line treatments with camrelizumab and apatinib was utilized to preliminarily assess the predictive performance of the model (24, 25).

In the safety analysis, we observed that apatinib monotherapy (750 mg) in aHCC patients was associated with hypertension incidences of 48% (all grades) and 28% (grade ≥3) (25). Notably, when combined with camrelizumab (250 mg), the incidence increased to 70% and 38%, respectively, suggesting a synergistic effect (9). To capture this key safety signal, we added a blood pressure dynamics module based on drug exposure levels of apatinib and camrelizumab, enabling quantitative prediction of hypertension risk. The model parameters were optimized using hypertension incidence data from apatinib monotherapy, and data from the combination with camrelizumab were used to validate the model’s predictive capability for hypertension occurrence.

### 2.3 Model application

#### 2.3.1 Exploration of biomarkers

The developed QSP model was employed to conduct a virtual clinical trial involving 500 patients. The treatment regimen comprised camrelizumab (200 mg, administered biweekly) combined with apatinib (250 mg, taken daily). According to the RECIST v.1.1 criteria, patients were classified as responders or non-responders. Informed by biomarker findings from other QSP models and subgroup analyses from clinical trials of the camrelizumab-apatinib combination, we identified seven key biomarkers relevant to the virtual patient cohort: initial tumor diameter, PD-L1 expression, CD8+ T cell density, Treg cell density, Macrophage density, CD4+/Treg ratio, and CD8+/Treg ratio (18, 26, 27). These biomarkers were investigated to evaluate their potential as indicators of treatment efficacy. The ORR and its 95% confidence intervals for various subgroups were plotted using R (version 4.2.3) and RStudio software (Posit, version 1.4.1717, Boston, MA, USA).

Subgroup analysis has inherent limitations in capturing complex interactions and nonlinear effects among variables. To address these limitations, we integrated machine learning techniques for covariate selection. The process involved several key steps: First, we applied least absolute shrinkage and selection operator (LASSO) regression for initial screening to identify potential biomarkers (28). Subsequently, we utilized these biomarkers to construct a random forest model, further quantifying their importance and predictive performance regarding treatment outcomes. We specifically focused on biomarkers identified in both the subgroup analysis and machine learning selection, considering them as key covariates. Receiver operating characteristic (ROC) curve analysis was performed on the selected key biomarkers to determine optimal cut-off values for predicting treatment response. This approach offers a more comprehensive understanding of the relationship between biomarkers and treatment outcomes, thereby providing stronger support for clinical decision-making.

LASSO regression incorporates an L1 regularization term in the loss function, which compels many regression coefficients to shrink towards zero. This feature selection method reduces model complexity and enhances predictive accuracy, particularly when addressing datasets with numerous irrelevant features. Furthermore, LASSO aids in alleviating issues of multicollinearity, which is prevalent in high-dimensional datasets. Furthermore, we employed Random Forest, a supervised machine learning technique, to evaluate the significance of the selected biomarkers in predicting patient responses to treatment (29). The model was implemented using Python (Python Software Foundation, version 2024.20.0, San Francisco, CA, USA). A parameter grid was established to optimize critical model parameters, including the number of decision trees and the maximum depth of the trees, thereby defining the hyperparameter search space.

#### 2.3.2 Optimization of dosing regimen

In real-world clinical studies on advanced hepatocellular carcinoma (HCC), camrelizumab and apatinib are frequently employed, either as monotherapy or in combination, at fixed doses. The standard dosing regimens are as follows: (1) Camrelizumab (200 mg every 2 weeks) plus apatinib (250 mg once daily); (2) Apatinib monotherapy: 750 mg once daily; (3) Camrelizumab monotherapy: 3 mg/kg every 3 weeks. During treatment, the dosing frequency of apatinib may be adjusted in response to adverse effects. Possible adjustments include: (4) Apatinib taken every other day; (5) Apatinib taken daily for 5 consecutive days, followed by a 2-day break; (6) Apatinib taken daily for 2 consecutive days, followed by a 2-day break. These modifications aim to mitigate adverse effects while preserving therapeutic efficacy. For other solid tumors, the combination regimen is as follows: (7) Camrelizumab (200 mg every 3 weeks) plus apatinib (250 mg once daily). To explore the feasibility of dose reduction and sequential therapy, we also investigated the following regimens: (8) No treatment; (9) Camrelizumab (200 mg every 2 weeks) plus apatinib (125 mg once daily); (10) Apatinib (250 mg daily for 7 consecutive days), followed by camrelizumab (200 mg every 2 weeks).

In addition to the virtual patients utilized in the CARES-310 clinical study, five additional cohorts, each comprising 500 patients, were generated. These cohorts were employed to simulate the aforementioned dosing regimen in order to evaluate its efficacy compared to the standard treatment plan. Efficacy was assessed every 8 to 12 weeks using RECIST v.1.1 criteria, with the aim of identifying the dosing regimen that offered optimal efficacy and minimal adverse effects (hypertension).

## 3 Results

### 3.1 Model development

**Figure 1:**
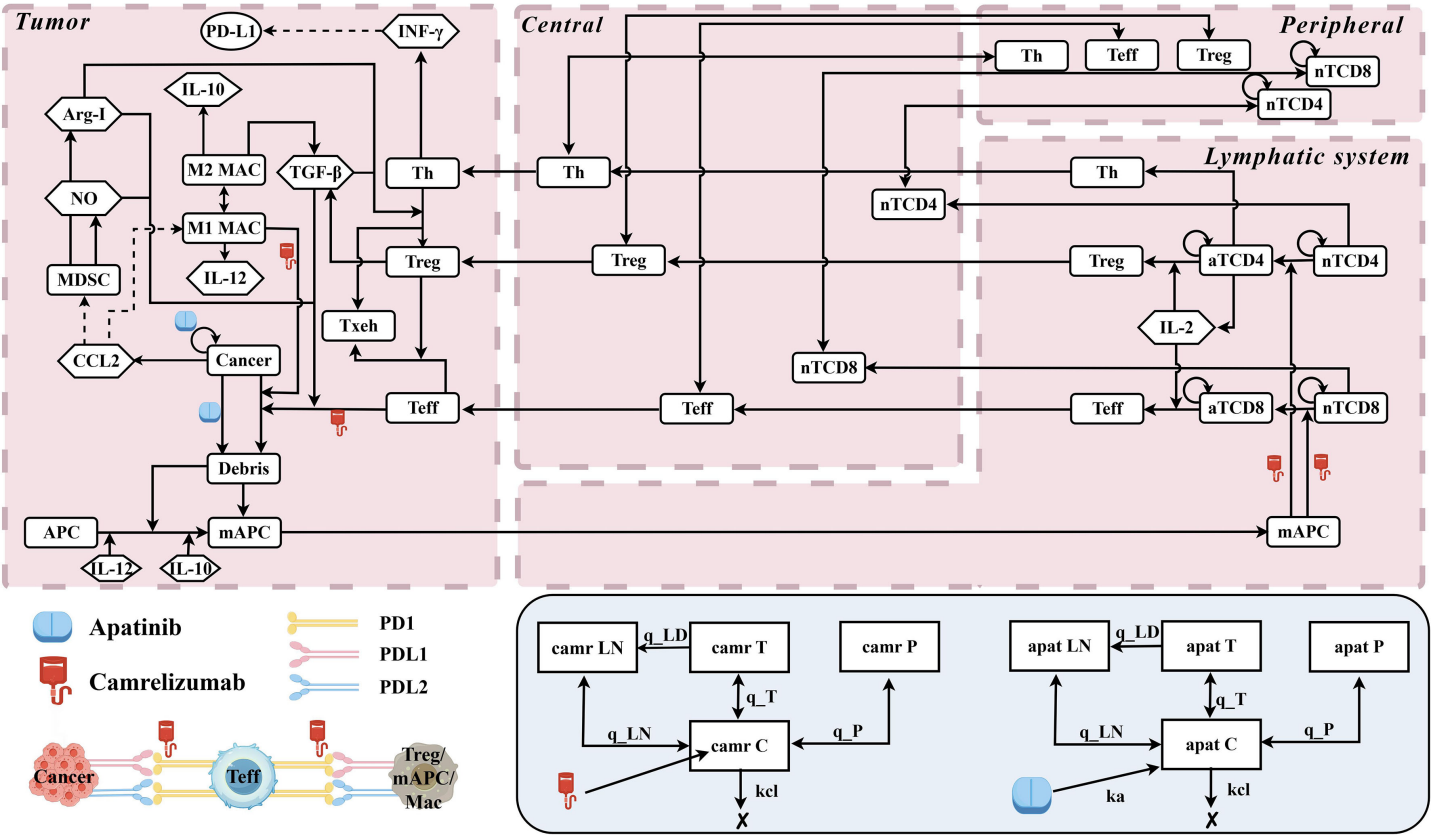
The dynamics of the major species in the quantitative systems pharmacology model of patients with advanced hepatocellular carcinoma. C, central compartment; P, peripheral compartment; LN, lymph node compartment; T, tumor compartment; kcl, elimination rate constant from central compartment; q_LN, transfer rate constant between central and LN compartments; q_T, between central and tumor compartments; q_P, between central and peripheral compartments; q_LD, between tumor and LN compartments; APC, antigen-presenting cell; mAPC, mature antigen presenting cell; nT, naïve T cell; aT, activated T cell; Teff, effector T cell; Texh, exhausted T cell; Th, T helper cell; Treg, regulatory T cell; MDSC, myeloid-derived suppressor cell; NO, nitric oxide; Arg-I, arginase I; camr, camrelizumab; apat, apatinib.

**Figure 1** illustrates the molecular and cellular interactions that govern the dynamics of various species within the model. The modular structure of the model in this study builds upon the QSP model previously established by Wang et al (30). In addition to incorporating the pharmacokinetic parameters of camrelizumab into the original model, we integrated the pharmacokinetics of apatinib, its tumor-killing effect, and its inhibition of tumor angiogenesis to develop a new apatinib module (31–33).

In the cancer compartment, it depicts processes such as cancer cell proliferation, the release of self- and cancer-associated antigens from dying cancer cells, macrophage recruitment and polarization, and the elimination of cancer cells by Teff. The activation of naïve CD8+ and CD4+ T cells by mature APCs (mAPCs) is shown in the lymph node compartment, followed by their differentiation into Teff and Treg, after which they are transported to both the central and peripheral compartments. Following intravenous administration of camrelizumab, the drug is transported to the cancer compartment, where it enhances the cytotoxic activity of Teff cells against cancer cells by binding to the PD-1 receptor. Concurrently, after oral administration, apatinib is absorbed into the central compartment, from where it is transported to the cancer compartment, where it exerts direct cytotoxic effects on cancer cells. Additionally, apatinib inhibits vascular endothelial growth factor receptor (VEGFR) activity, thereby blocking tumor vasculature growth. This QSP model comprehensively accounts for the impact of these drugs on the tumor cell population, with the corresponding computational equations provided in Equation 1:

(1) $$\begin{array}{l} \frac{dC}{dt}=k_{\text{g}rowth}\times C\times \log\left(\frac{C_{\text{t}otal}}{C_{max}}\right)\\ \;-C\times \left[k_{innate}+k_{apat}\times \frac{\left[APAT\right]}{\left[APAT\right]}\times f_{apat}\right]\\ \;-C\times \left[k_{T_{cell}}\times \frac{T}{\text{T+}k_{C}\times C_{\text{total}}}\times \frac{T}{\text{T+}k_{T_{reg}}\times C_{T_{reg}}}\times \left(1-H_{PD1}\right)\times \left(1-H_{TGF}\right)\times \left(1-H_{MDSC}\right)\right]\\ \;-C\times \left[k_{M1}\times \frac{M1}{M1+k_{Mac}\times C}\times \left(1-H_{Mac}\right)\times \left(1-H_{IL10}\right)\right] \end{array}$$

Here, *C* represents the number of cancer cells, and *k_growth_* is the growth rate constant for cancer cells. *C_total_* denotes the total number of cancer cells in the tumor compartment, while *C_max_* is the maximum carrying capacity for cancer cells in the tumor. *k_innate_* is the death rate constant for cancer cells induced by the innate immune system, and *k_apat_* represents the rate constant for the inhibition of cancer cell proliferation by apatinib. The concentration of apatinib in the tumor compartment is denoted as *[APAT]*, and *IC50_apat_* refers to the concentration of apatinib required to inhibit 50% of cancer cell proliferation. *f_apat_* is the uptake rate of apatinib by cancer cells, and *k_Tcell_* represents the rate constant for cancer cell death induced by T_eff_. *k_C_* is the maximum killing rate of cancer cells by Teff cells. *k_Treg_* is the rate constant for the inhibition of cancer cell proliferation by T_reg_, and *C_Treg_* denotes the number of T_reg_ in the tumor compartment. *T* represents the number of effector T cells. The model also incorporates the inhibitory effects of PD-1 immune checkpoint (*H_PD1_*), TGF-β (*H_TGF_*), and MDSC(*H_MDSC_*) on T cell activity. *k_M1_* represents the rate constant for the phagocytosis of cancer cells by M1 macrophages, and *M1* is the number of M1 macrophages. *k_Mac_* quantifies the dependence of the phagocytosis rate on the ratio of M1 macrophages to cancer cells. Finally, the inhibitory effects of signal regulatory protein alpha (SIRPA, *H_Mac_*) and IL-10 (*H_IL10_*) on macrophage phagocytosis are included in the model. Cmax is calculated using Equation 2.

(2) $$\frac{dC_{max}}{dt}=k_{K,g}\times C_{total}\times \frac{C_{vas}}{C_{vas}+EC50_{vas}}-k_{K,d}\times C_{max}\times {\left(C_{total}\times V_{cell}\right)}^{\frac{2}{3}}-k_{K,apat}\times C_{max}\times [APAT]$$

*k_K,g_* represents the baseline rate of tumor angiogenesis driven by angiogenic factors. *C_vas_* denotes the effective concentration of these factors during the tumor vascularization process. *EC_50vas_* is the half-maximal concentration required to elicit a vascular response mediated by angiogenic factors. *k_K,d_* characterizes the endogenous inhibition rate of vascular growth, which is regulated by mechanisms such as endothelial cell apoptosis. *V_cell_* corresponds to the volume of an individual aHCC cell. *k_K,apat_* quantifies the inhibitory effect of apatinib on tumor angiogenesis.

In the blood pressure dynamics module for apatinib and camrelizumab exposure, the relationship between drug concentration and blood pressure changes is described by Equation 3:

(3) $$\begin{array}{l} blood\_pressure=E_{BPbase}+\frac{BP\_E_{\max}\times C\_Capat^{BP\_HIll}}{BP\_EC_{50}+C\_Capat^{BP\_HIll}}\\ \;+\frac{BP\_E_{\max}\times C\_Capat^{BP\_HIll}}{BP\_EC_{50}+C\_Capat^{BP\_HIll}}\times C\_Ccamr\times f\_BPcamr \end{array}$$

Here, *E_BPbase_* represents the baseline blood pressure of the patient before treatment, *BP_E_max_* is the maximum blood pressure increase induced by apatinib, *BP_EC_50_* is The half maximal effective concentration (EC_50_) of apatinib-induced hypertension, *C_C_apat_* is the central compartment concentration of apatinib, *BP_Hill* is the Hill coefficient for apatinib-induced hypertension​, *C_C_camr_* is the central compartment concentration of camrelizumab, and *f_BPcamr* is the coefficient describing the synergistic effect of camrelizumab on apatinib-induced blood pressure elevation. Blood pressure of 120–159 mmHg was classified as grade 1/2 hypertension, while ≥160 mmHg was classified as grade 3/4 hypertension. To avoid the occurrence of outliers, the maximum predicted blood pressure value was set to 220 mmHg.

To accurately reflect the distribution of real clinical patient characteristics, the model parameter subsets of the virtual patient queue are varied (refer to **Supplementary Table S1**). The baseline values and ranges for the population characteristic parameters are sourced from both experimental and clinical data, as outlined in **Supplementary Table S2**. Additional parameters, species, and the rules governing the model are detailed in the **supplementary materials** of Wang et al.’s article (34). For certain parameters without reported experimental values (**Supplementary Table S2**), fitting was performed based on clinical trial results in aHCC patients treated in CARES-310 or with apatinib/camrelizumab monotherapy. In this study, sensitivity analysis was conducted only for variables with assigned variability.

In this study, we developed a virtual patient model comprising 500 patients to simulate tumor treatment responses. During initialization, 10.4% of patients failed to reach the expected baseline tumor size, and 6.4% showed parameter non-convergence. During model execution, we noted that the parameter values for a subset of virtual patients (10 out of 416) exceeded the pre-established reasonable range (refer to **Supplementary Table S3**). The characteristics of these non-target virtual patients (94/500) are presented in **Supplementary Table S4**, with t-tests conducted to compare them with the complete dataset.

**Figure 2:**
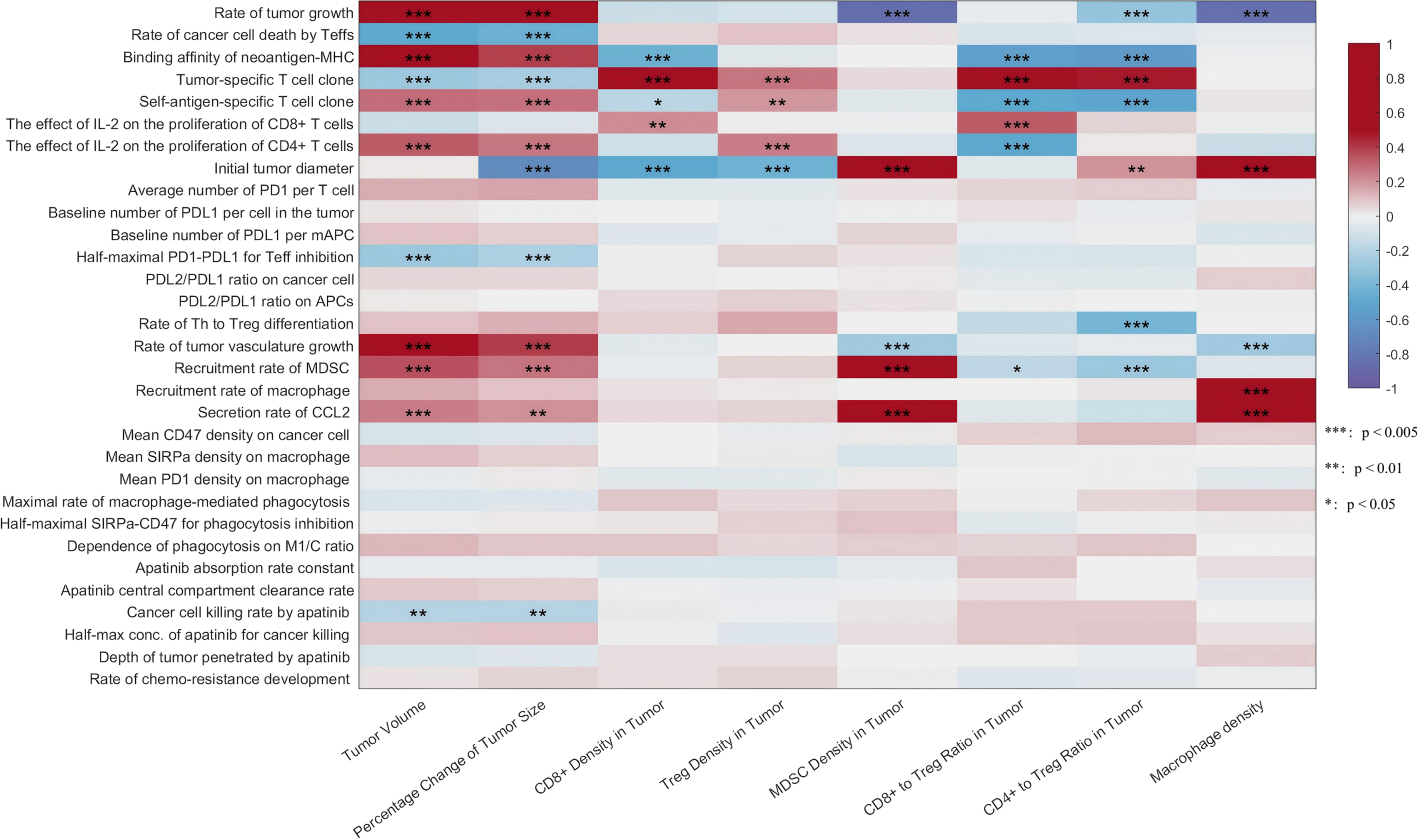
Global uncertainty and sensitivity analysis were conducted using Latin Hypercube Sampling (LHS) to assign thirty-one parameters based on our estimated distribution. The Partial Rank Correlation Coefficient (PRCC) between selected post-treatment observations and input parameters is presented in the form of a heatmap. ***, extremely significant; **, highly significant, *, Significance.

To further investigate the correlation between modeling parameters and key outcome indicators, such as tumor volume, we conducted a sensitivity analysis using the PRCC (**Figure 2**). The results indicated a significant positive correlation between tumor growth rate, neoantigen-MHC binding affinity, and tumor angiogenesis rate with tumor volume. Additionally, the tumor’s initial diameter, the recruitment rate of MDSCs, and the level of C-C motif chemokine ligand 2 (CCL2) demonstrated a significant positive correlation with MDSC cell density in the tumor. Notably, a strong negative correlation was identified between tumor growth rate and the cell density of MDSCs and macrophages.

### 3.2 Model evaluation

**Figure 3:**
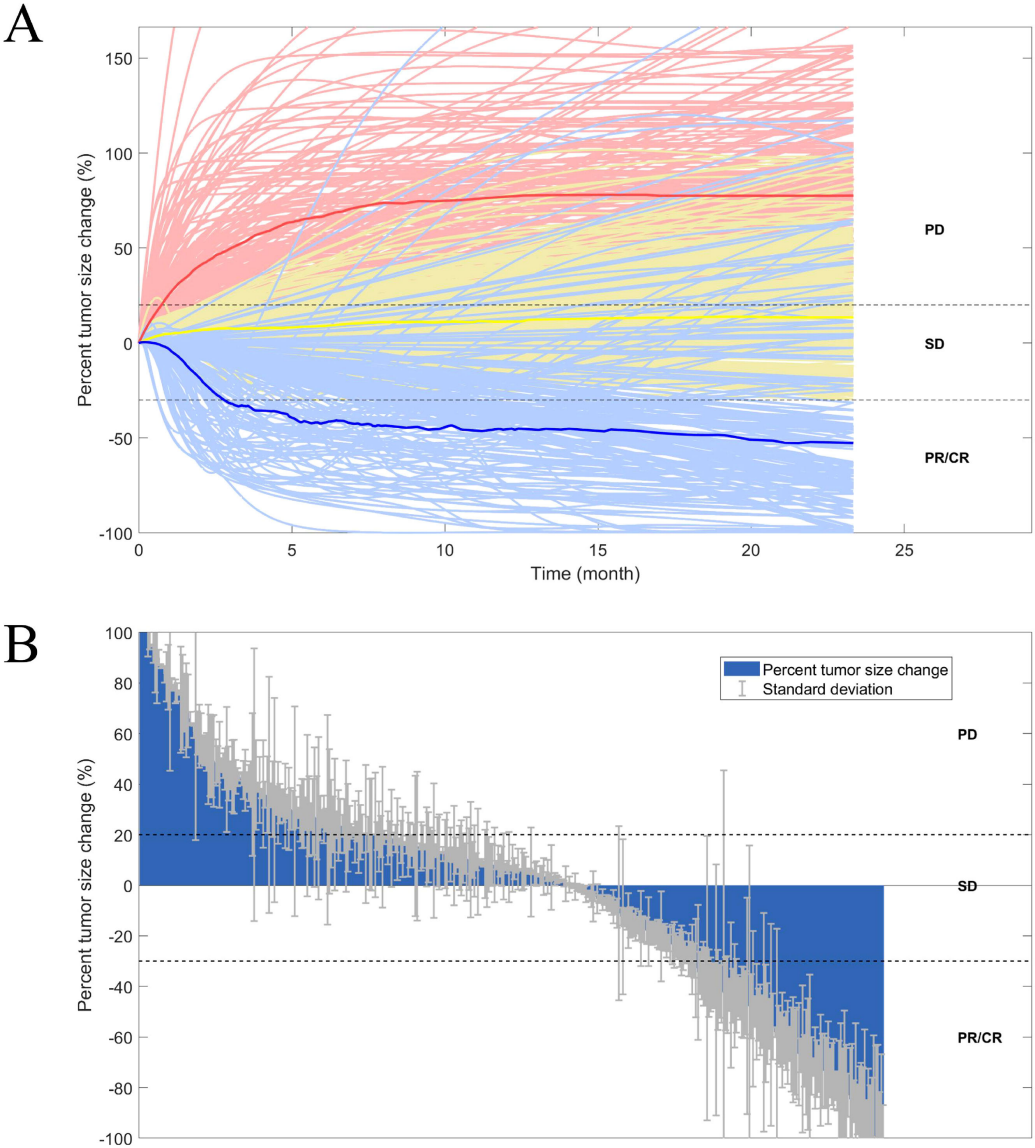
Rate of response **(A)** and the best overall response **(B)** in model-predicted tumor diameter of 406 randomly virtual patients. Response is assessed by RECIST V.1.1. Median (thick lines) and individual (thin line) rate of response are shown in PD (red), SD (yellow), and PR/CR (blue) subgroups. Dashed lines correspond to +20% and −30%. CR, complete response; PD, progressive disease; PR, partial response; SD, stable disease.

In **Table 1**, we present a comparison of the simulation results derived from our model for 406 reasonable virtual patients against actual data obtained from the CARES-310 clinical trial. The ORR following treatment with standard doses of camrelizumab (200 mg, intravenously administered biweekly) in conjunction with apatinib (250 mg, orally administered once daily) was found to be 25%, while the median DOR was recorded at 14.9 months. The difference between these predictive values and the results of the CARES-310 clinical trial is within 5%, and it closely aligns with the ORR observed in clinical studies of non-first-line treatments using camrelizumab or apatinib monotherapy. These findings validate the accuracy and reliability of our model’s predictions. Additionally, **Figure 3** illustrates the time-varying response and the waterfall plot of tumor dynamics, which align with the findings observed in the CARES-310 study, further corroborating the reliability of the QSP model we have established. As shown in **Supplementary Table S3**, the deviation between our model predictions and the observed hypertension incidence in the CARES-310 clinical trial was 6.14%, demonstrating the model’s robust predictive performance.

**Table 1:** Efficacy prediction for the virtual patient cohort generated based on calibrated parameter distribution.

Efficacy measures	Camrelizumab Plus Apatinib		Camrelizumab monotherapy (3 mg/kg q2w)		Apatinib monotherapy (750 mg qd)	
	Observation (95% CI)	Prediction (95% CI)	Observation (95% CI)*	Prediction (95% CI)	Observation (95% CI)*	Prediction (95% CI)
n	272	406	109	436	261	418
ORR (%)	25.4 (20.3 - 31.0)	25.4 (21.1 - 29.6)	11.9 (6.5 - 19.5)	13.5 (10.3 - 16.8)	10.7 (7.0 - 15.0)	10.8 (7.8 - 13.8)
Median DOR (month)	14.8 (8.4 - NR)	14.9 (11 - NR)	NE	NE	NE	NE

ORR, objective response rate; DOR, duration of response; NR, not reached; NE, not evaluated; *, second-line or later therapy; q2w, biweekly; qd, Once a day.

### 3.3 Model application

#### 3.3.1 Exploration of biomarkers

**Figure 4:**
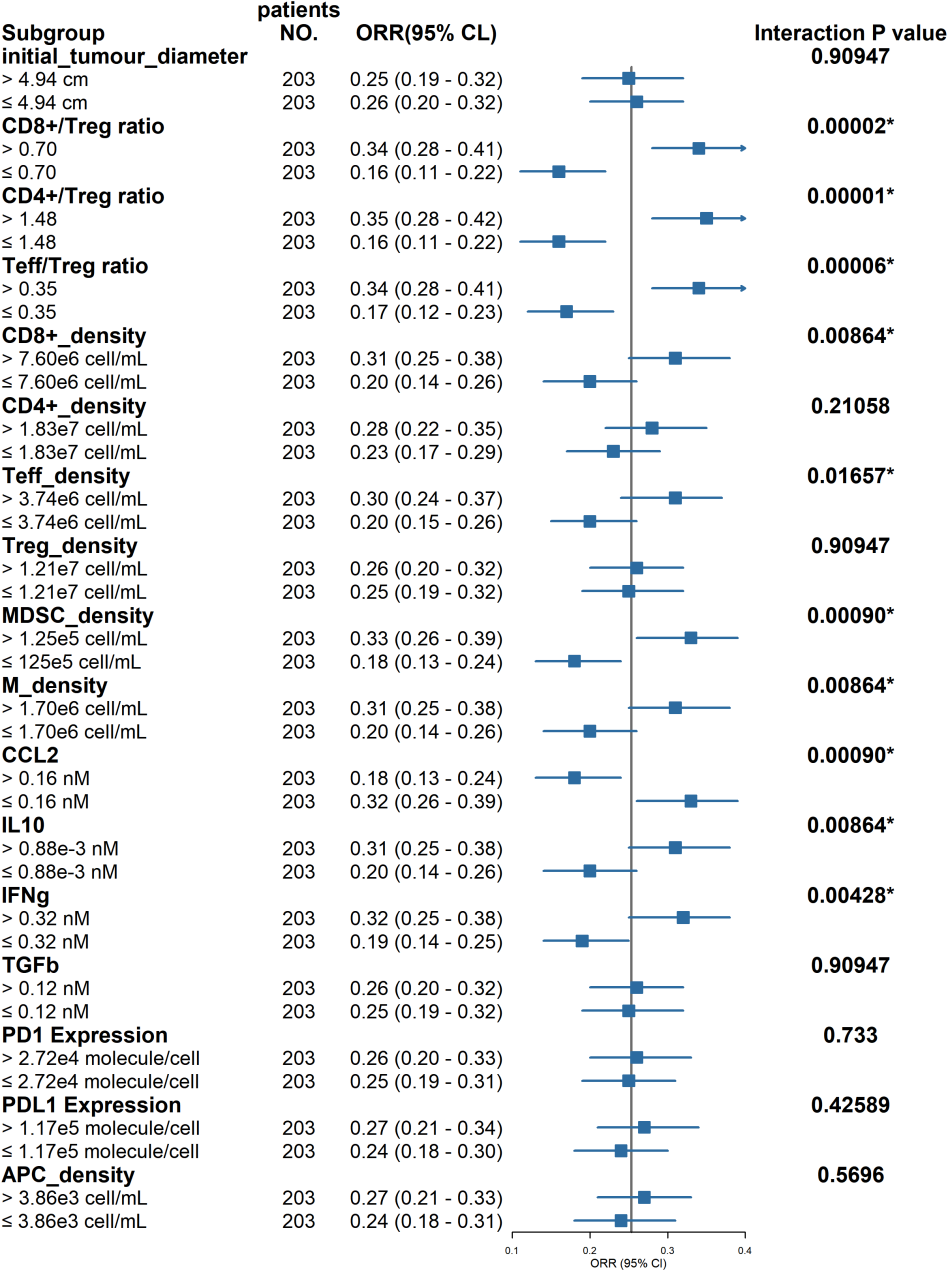
Subgroup analysis of the combination therapy. Virtual patients (N = 406) are divided into 14 subgroups based on the pretreatment values of selected biomarkers. Objective response rates (ORR) for each group are given along with the 95% CI estimated. The black solid line indicated the ORR for the total population. Groups were divided by median values; Interaction p value indicates whether two or more variables show significant interaction (p<0.05); initial_tumour_diameter, baseline tumor diameter; PD1 expression, average PD1 per T cell; PDL1 expression, baseline PDL1 per tumor cell; TGFb, transforming growth factor-β; MDSC, myeloid-derived suppressor cell; M, macrophage; IL10, interleukin 10; IFNg, interferon-γ; CCL2, C-C motif chemokine ligand 2.

This study further investigates seven measurable biomarkers through subgroup analysis to identify potential predictors of patient response to treatment. **Figure 4** presents the ORR and the corresponding confidence intervals for patient groups stratified by the median values of each biomarker. This analysis identifies several key factors influencing the ORR in combination therapy, including the Teff/Treg ratio, MDSC density, macrophage density, interleukin-10 (IL-10) concentration, interferon-gamma (IFN-γ) concentration, CCL2 concentration, CD8+/Treg ratio, and CD4+/Treg ratio.

**Figure 5:**
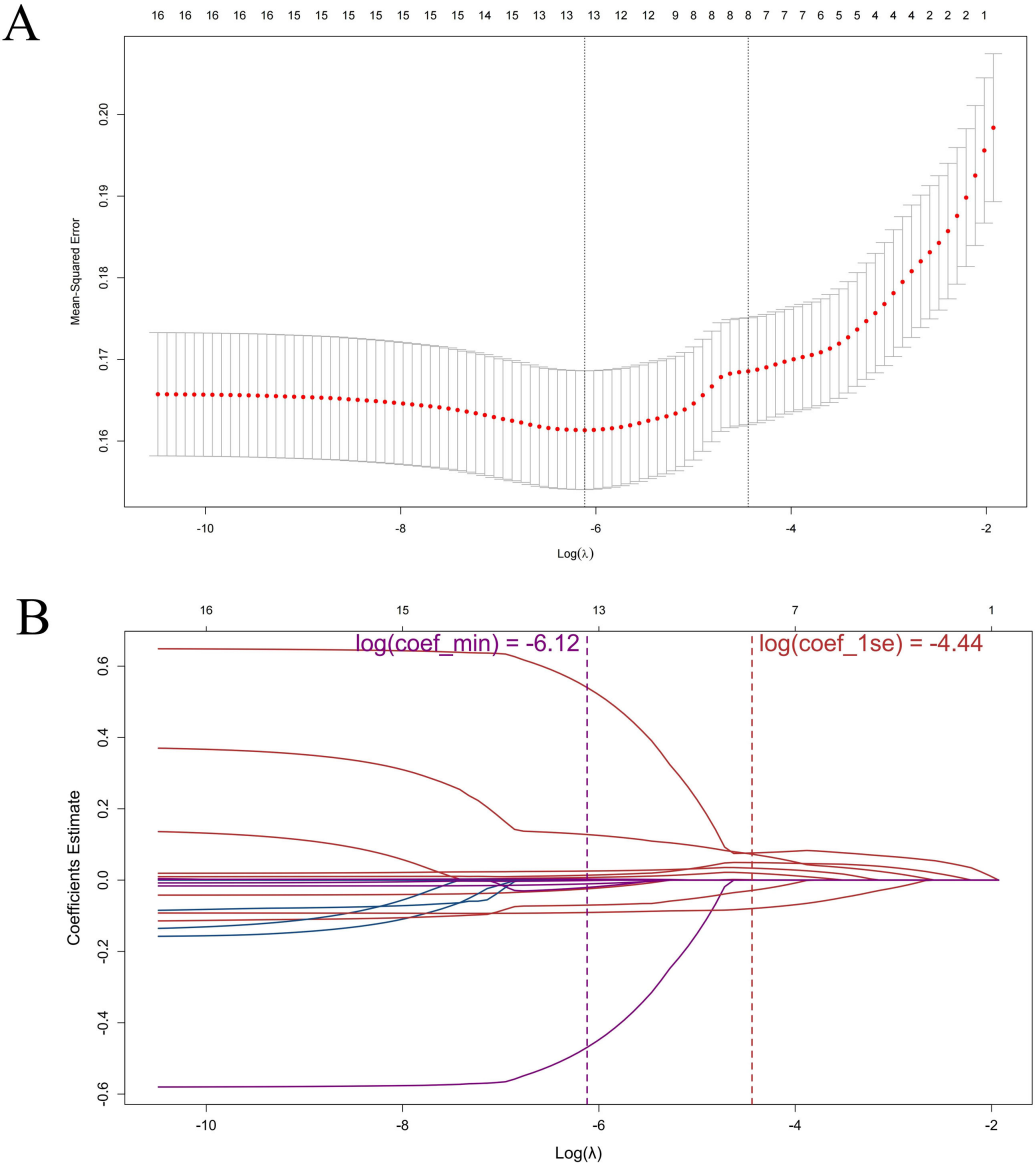
Selection of significant parameters in the clinicopathologic from variables in the training set. **(A)** Ten-fold cross-validation for tuning parameter selection in the LASSO model. The solid vertical lines represented the binomial deviance ± standard error (SE). The dotted vertical lines were drawn at the optimal values using the minimum and 1-SE criteria. **(B)** LASSO coefficient profiles. The LASSO shrank some regression coefficients to exactly zero. The binomial deviance curve was plotted versus log (λ), where λ was the tuning parameter. LASSO, Least Absolute Shrinkage and Selection Operator; coef_min, λ giving minimum cross-validation error; coef_1se, largest λ within one standard error of the minimum.

**Figure 6:**
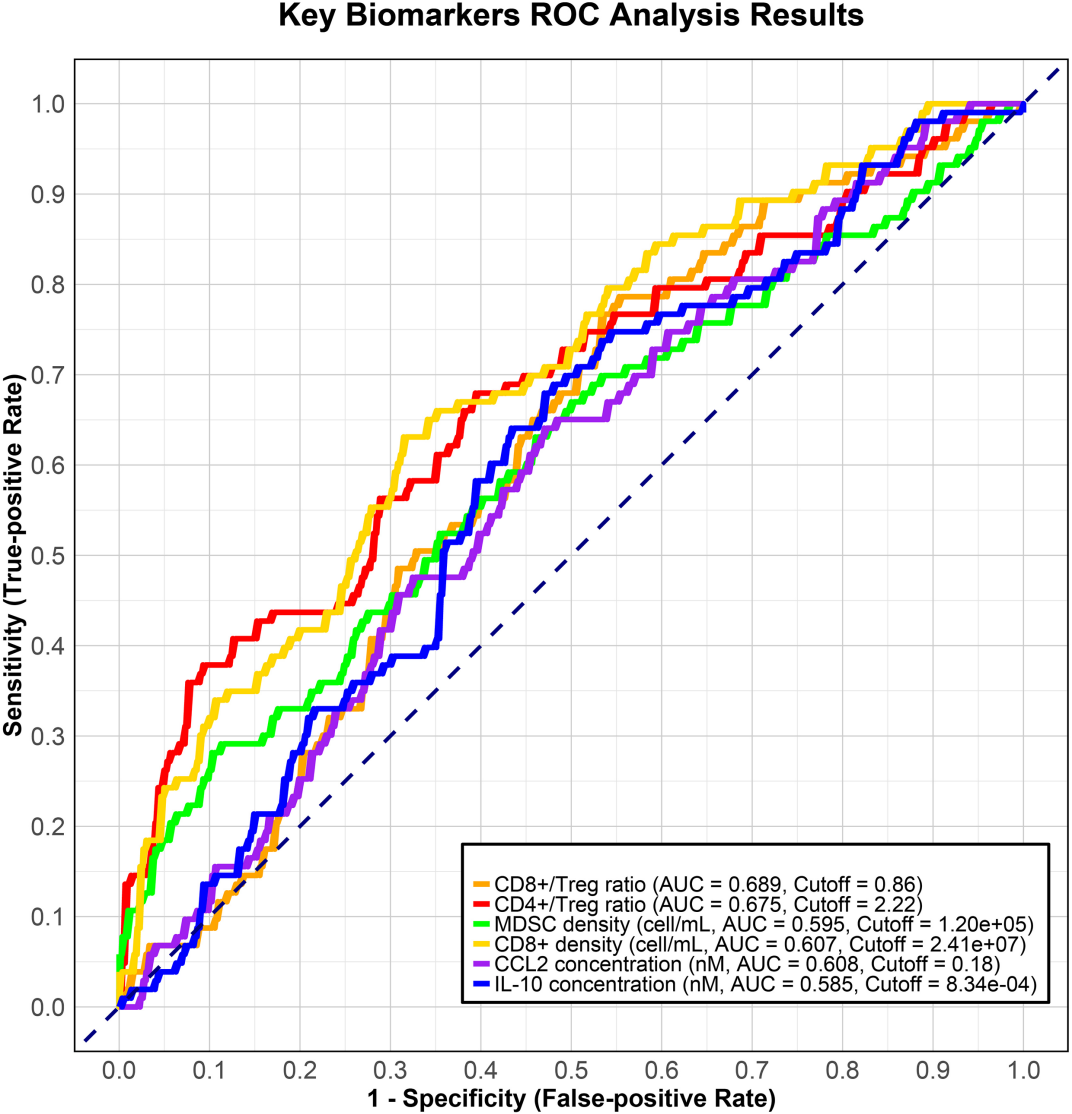
Key Biomarkers ROC Analysis Results. ROC, receiver operation characteristic curve; MDSC, myeloid-derived suppressor cell; CCL2, C-C motif chemokine ligand 2; IL10, interleukin 10; AUC, areas under curve.

The results of the LASSO regression analysis are depicted in **Figure 5A**, which illustrates the relationship between the binomial deviance curve and the logarithm of the tuning parameter λ (log(λ)). The optimal λ value was identified through the analysis of the deviance curve, yielding an optimal value of 0.012, which corresponds to log(λ) = -4.44. Utilizing this optimal λ, we subsequently generated the coefficient profile, as presented in **Figure 5B** and **Table 2**. LASSO regression analysis identified the CD8+/Treg ratio, CD4+/Treg ratio, MDSC density, CD8+ density, CCL2 concentration, IL-10 concentration, Treg density, and initial tumor diameter as variables of interest. The first six parameters were consistent with the results of the subgroup analysis and are therefore considered biomarkers closely associated with therapeutic efficacy. ROC analysis of these parameters, shown in **Figure 6**, indicated that the CD8+/Treg ratio had the highest AUC, with a cut-off value of 0.86.

**Table 2:** The coefficients of variables in LASSO.

Variables	Coefficients
initial_tumour_diameter	0.0007
CD8+/Treg ratio	0.0761
CD4+/Treg ratio	0.0491
CD8+_density	0.0727
Treg_density	-0.0289
MDSC_density	0.0336
CCL2	-0.0797
IL10	0.0195

MDSC, myeloid-derived suppressor cell; CCL2, C-C motif chemokine ligand 2; IL10, interleukin 10.

**Figure 7:**
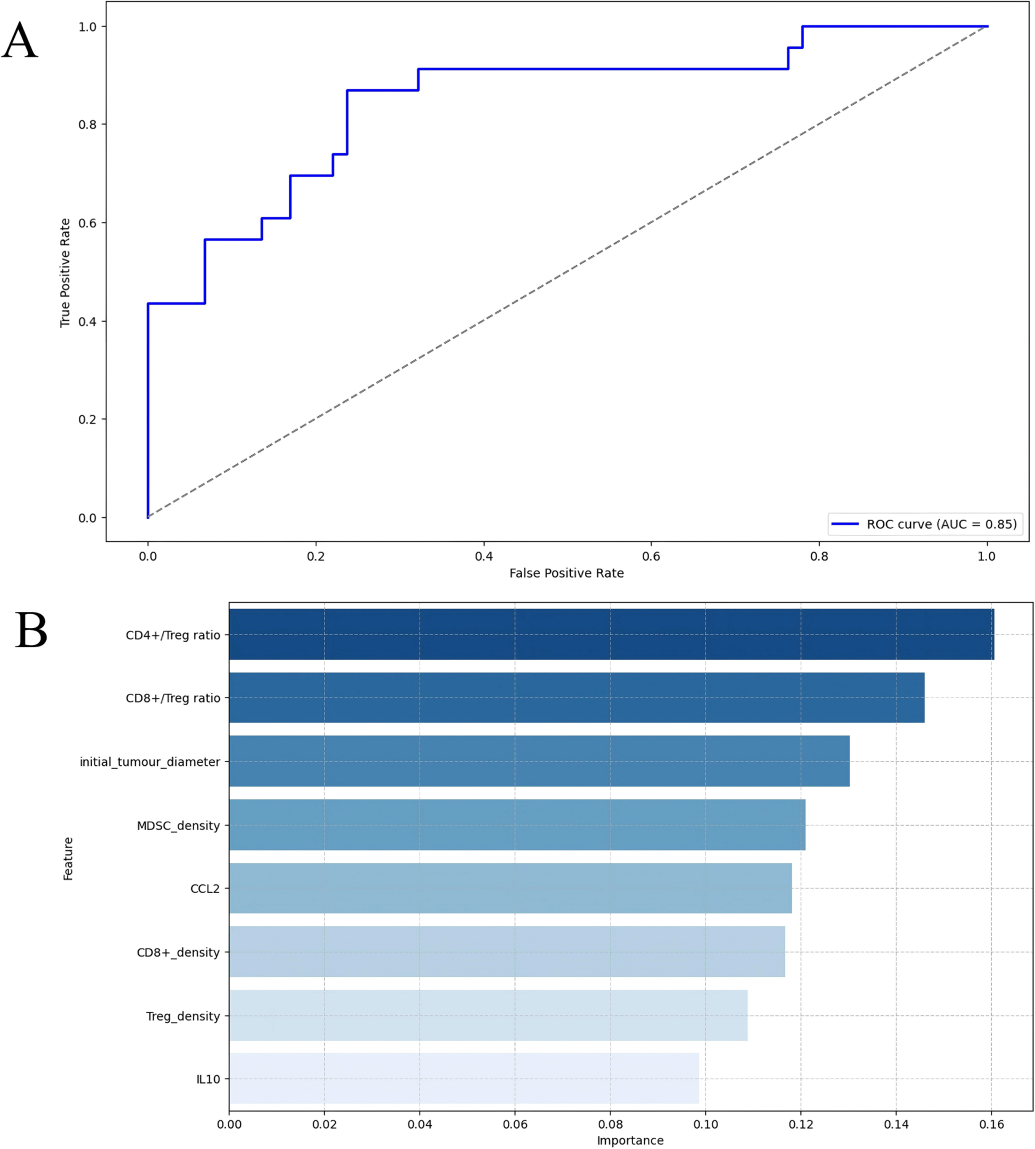
Exploring biomarkers using a random forest classifier. **(A)** Receiver operation characteristic curve (ROC) of random forest classifier. The dashed line represents the performance of the random classifier, which is the ideal classifier without classification ability. **(B)** The importance of features in random forest classifier. AUC, areas under curve; TGFb, transforming growth factor-β; MDSC, myeloid-derived suppressor cell; M, macrophage; IL10, interleukin 10; IFNg, interferon-γ; CCL2, C-C motif chemokine ligand 2; initial_tumour_diameter, baseline tumor diameter.

Using simulation results from 406 virtual patients in the combination therapy group, we developed a random forest model comprising 5000 trees, utilizing 9 biomarkers (derived from LASSO screening results) as predictor variables. The model exhibited robust predictive performance, achieving accuracies of 0.94 on the training set and 0.80 on the test set. **Figure 7A** presents the Receiver Operating Characteristic (ROC) curve of the random forest classifier, further validating the model’s strong classification capability. Additionally, **Figure 7B** illustrates the importance of each biomarker within the model. The results indicate that, prior to treatment, the CD4+/Treg ratio and initial tumor diameter are crucial biomarkers closely associated with patient response to treatment.

#### 3.3.2 Optimization of dosing regimen

To evaluate the potential of the current QSP platform for dose optimization, we designed and conducted ten distinct virtual clinical trials utilizing existing virtual patient datasets. The detailed results of the efficacy evaluations conducted at 8-week and 12-week intervals for each clinical trial group are presented in **Table 3**. Our analysis considered both ORR and median DOR, revealing that synchronous combination therapy demonstrated a slight advantage over sequential therapy. To optimize the dosing frequency of apatinib, we compared various dosing regimens. Research has demonstrated that, in addition to daily dosing regimens, a continuous daily dosing regimen lasting five days followed by a two-day cessation also exhibits comparable ORR. Additionally, we observed comparable ORR at doses of 250 mg and 125 mg of apatinib, while the dose reduction from 250 mg to 125 mg led to a 25% (0%–56%) decrease in all-grade hypertension and a 27% (0%–61%) decrease in grade ≥3 hypertension (**Supplementary Table S6**), highlighting its potential to mitigate apatinib-associated toxicity.

**Table 3:** Prediction of therapeutic efficacy under different administration strategies.

Medication strategy		Efficacy prediction	
Camrelizumab	Apatinib	ORR (%)	Median DOR (month)
*200 mg q2w	250 mg qd	26.0 (23.8-28.1)	14.0 (11.2-15.9)
NA	750 mg qd	11.4 (10.3-12.5)	13.5 (12.1-14.9)
3 mg/kg q3w	NA	13.0 (12.4-13.5)	18.4 (16.8-18.7)
200 mg q2w	250 mg qod	22.2 (19.6-25.2)	13.8 (12.1-14.9)
200 mg q2w	250 mg qd × 5, then 2-day hiatus	24.4 (22.4-28.0)	12.8 (11.2-13.1)
200 mg q2w	250 mg qd × 2, then 2-day hiatus	21.9 (19.2-24.6)	14.8 (14.0-14.9)
200 mg q3w	250 mg qd	25.2 (23.1-27.6)	13.7 (11.2-14.9)
200 mg q2w	125 mg qd	24.4 (21.5-26.8)	13.1 (11.2-14.9)
200 mg q2w began on the seventh day	250 mg qd	25.6 (23.8-27.6)	13.1 (11.2-14.9)
NA	NA	NA	NA

ORR, objective response rate; DOR, duration of response; NA, Not Applicable; *, standard treatment plan; q2w, biweekly; q3w, once every three weeks; qd, once a day; qod, once every other day.

## 4 Discussion

In the past decade, although systemic treatment for aHCC has progressed, new therapeutic strategies continue to encounter challenges such as low response rates and drug resistance (35). Utilizing the QSP-IO platform developed by Wang et al. and previously reported pharmacodynamic and pharmacokinetic parameters, we established a QSP model for aHCC patients undergoing the camrelizumab plus apatinib regimen. By generating virtual patients to simulate the CARES-310 trial, the model’s predicted ORR and median DOR were highly consistent with clinical observations. This consistency demonstrates the potential of virtual clinical trials in identifying key biomarkers, optimizing treatment plans, and providing robust support for the screening of advantageous populations and the customization of individualized dosing regimens for camrelizumab combined with apatinib treatment. Compared with a previous QSP model of aHCC patients, our model incorporated more parameters (279 vs. 140), which partly explains the higher rate of non-convergence (6% vs. 3%) (19). Each virtual patient was simulated starting from a single tumor cell, and the initial parameter set required to achieve the preset initial tumor volume was recorded as that of a standard virtual patient. We found no strong correlation between tumor volume and initial tumor diameter, contrary to the findings of Wang et al.’s study (36). Building upon the original QSP model, we incorporated an apatinib module to characterize the pharmacokinetic process of apatinib in the body and its effects on tumor cells. As demonstrated in the sensitivity analysis, the inclusion of the apatinib module did not result in an underestimation of the effect of immunotherapy. This is attributed to the fact that the efficacy of the combination therapy was significantly related not only to the parameters associated with apatinib but also to other immune parameters. Another interesting observation was the negative correlation between tumor growth rate and MDSC/macrophage density in the sensitivity analysis (**Figure 2**). Mechanistically, although a higher tumor growth rate enhances CCL2 secretion and promotes immune cell recruitment, the rapid expansion of tumor volume outpaces this recruitment, ultimately leading to a decrease in relative immune cell density. This finding underscores the importance of distinguishing between absolute numbers and relative density when analyzing the tumor microenvironment. Constructing virtual cohorts that closely match target populations is essential before conducting virtual clinical trials. However, developing reliable oncology virtual patients remains challenging due to limited clinical data in public databases and the heterogeneity of trial-recruited cohorts (37). To address this, researchers have designed screening methods based on probability density matching or parameter space mapping algorithms to align with clinical data distributions (38, 39). However, the complexity of QSP models and strong parameter correlations can introduce biases or significantly increase computational costs. To balance model applicability and computational efficiency, this study employed LHS for parameter generation and filtered virtual patients (VPs) using predefined feasibility thresholds.

In the biomarker analysis, we conducted a subgroup analysis to investigate significant variables associated with treatment response. For the combination therapy of camrelizumab and apatinib, our model confirmed that the CD8+/Treg ratio, CD4+/Treg ratio, and Teff/Treg ratio were the most predictive biomarkers, as they exhibited a higher ORR in the high-expression subgroup. Studies have demonstrated that the CD8+/Treg ratio in tumors of elderly mice is lower than that in their younger counterparts (40). This observation may elucidate the findings of the CARES-310 clinical study, which indicates that patients under the age of 65 derive greater benefits from combination therapy compared to those aged 65 and older (9). Furthermore, the model identified that the high CD8+ cell subgroup could achieve superior efficacy, aligning with the biomarkers reported for this combination therapy regimen in other solid tumors (41). However, discrepancies were noted between the model-predicted biomarkers and clinical observations. As depicted in **Figure 4**, the pretreatment PD-L1 expression level was not as closely correlated with efficacy as indicated in other studies, potentially due to Xu et al.’s choice of progression-free survival (PFS) as the efficacy endpoint (26). A similar situation was observed with APC cell concentration, where the disparity may be attributed to differences in immune biology between patients with resectable and unresectable hepatocellular carcinoma (42). In recent years, machine learning algorithms have gained significant traction in clinical decision-making (43). Unlike traditional methods, machine learning does not require the assumption of key drivers to infer or identify unknown associations. However, the ‘black box’ nature of these algorithms often results in outcomes that lack mechanistic explanations (44). The QSP model, grounded in complex mechanisms, addresses this limitation by providing a more comprehensive understanding. Furthermore, the availability of clinical and preclinical data remains limited. The QSP model can generate extensive datasets that are challenging to obtain using conventional technologies. Currently, an increasing number of studies are exploring the integration of QSP models with machine learning techniques (45). For instance, Coletti et al. developed a QSP model for prostate cancer immunotherapy and constructed a decision tree to analyze the results, thereby predicting the potential causality of various treatment regimens (46). In the realm of liver cancer, Lasso regression has emerged as a commonly employed algorithm, particularly for predicting patients’ recurrence risks and identifying biomarkers related to drug treatment efficacy (47).

To further identify individuals who may benefit from tailored treatment plans and to develop personalized diagnostic and therapeutic strategies for advanced liver cancer patients, we conducted LASSO regression analysis on 17 parameters of virtual patients prior to treatment administration. This analysis revealed 8 biomarkers closely associated with treatment response status, including the CD8+/Treg ratio and CCL2 concentration in tumors (see **Figure 5**, **Table 2**). Research indicates that CCL2 is overexpressed in human liver cancer and is closely linked to patient prognosis (48). Previous work by Xia et al. demonstrated that combining PD-L1 blockade with FGFR4 or MAPK inhibition significantly reduces CCL2 levels and suppresses metastatic progression (49). Informed by these findings and our QSP modeling results, we hypothesize that incorporating CCL2/CCR2 blockade into camrelizumab plus apatinib therapy may alleviate myeloid-driven immunosuppression and enhance antitumor immune activity, thereby providing a mechanistically grounded strategy for personalized combination treatment in aHCC. Subsequently, the variables selected through LASSO regression were utilized to construct a random forest model. Typically, an area under the curve (AUC) of ≥ 0.7 signifies sufficient predictive capability (50). Our findings suggest that the random forest model established with the 8 biomarkers demonstrates strong predictive performance for the test set (AUC = 0.85). Notably, our research identified that the CD8+/Treg ratio, CD4+/Treg ratio, and initial tumor diameter significantly contribute to the random forest model. The six biomarkers identified through the combination of subgroup analysis and machine learning in this study show promising clinical applicability. In 2025, the European Society for Medical Oncology (ESMO) guidelines recommended histopathological confirmation of aHCC prior to initiating systemic therapy (51). This recommendation provides an excellent opportunity for biomarker testing, as residual biopsy samples can be quantitatively analyzed using immunohistochemistry, flow cytometry, and other techniques (51–53). Integrating the quantitative cut-off values identified in this study into existing clinical workflows may offer a direct and feasible reference for developing individualized treatment strategies, thereby facilitating the clinical translation of these biomarkers. Biomarker exploration using machine learning on QSP model outputs in aHCC has been reported previously, but unlike that study, our random forest model did not include PD-L1 expression (19). Our model, based on eight biomarkers, achieved an AUC of 0.85, indicating strong predictive performance and potential clinical utility. When PD-L1 expression was incorporated into the model, the AUC decreased to 0.79. This difference reflects variations in drug mechanisms and calibration trials. Notably, in the CARES-310 trial, PD-L1 was also not significantly associated with combination therapy efficacy, supporting our findings. Rather than acting as a determinant of response, PD-L1 appears to reflect adaptive immune evasion; its expression is both temporally dynamic and spatially heterogeneous (54, 55). In hepatocellular carcinoma, multiple concurrent immunosuppressive circuits—including MDSCs, tumor-associated macrophages, chemokine signaling, and angiogenic pathways—can attenuate the effects of PD-1/PD-L1 blockade, thereby limiting the predictive utility of PD-L1 as a standalone biomarker (56). Consistently, QSP-IO simulations indicate that PD-L1 levels do not capture overall immune competence, whereas mechanistic indices integrating effector and suppressive components (such as CD8+/Treg or CD4+/Treg ratios) better account for inter-patient heterogeneity in response to combination therapy. Our model achieved 80% accuracy in the test set, slightly lower than the 91.3% reported previously, likely due to differences in sample sizes (training set: n=325 vs. n=1365; test set: n=81 vs. n=50) (19). Nevertheless, the clinical utility of these biomarkers requires confirmation in large-scale real-world cohorts. Future studies will focus on collecting patient data from our center to prospectively or retrospectively evaluate their predictive performance and robustness, thereby providing a foundation for their integration into clinical decision-making.

During the dose simulation process, we explored not only the existing clinical trial regimen but also the feasibility of reducing the dose or frequency of administration. The simulated 750 mg apatinib monotherapy yielded an ORR consistent with that observed in the AHELP clinical trial (25). Furthermore, the ORR of camrelizumab monotherapy in the second-line treatment clinical trial (11.9% vs. 13.5%) closely aligned with our simulation results (24). Reducing apatinib to 125 mg achieved efficacy comparable to the standard dose (ORR 25.9% vs. 24.4%; median DOR 14.0 vs. 13.0 months) while lowering the incidence of hypertension by more than 25%. Prior evidence suggests that anti-angiogenic treatment at an appropriate dose range is sufficient to achieve vascular normalization and facilitate immune infiltration (57). Increasing the dose beyond this range may fail to enhance immunologic synergy and instead intensify hypoxia and toxicity. In contrast, lower dosing has been proposed to extend the duration of vascular normalization, thereby augmenting the therapeutic benefit of immunotherapy (58). Consistently, QSP simulations suggest that the biologically effective dose of apatinib required for immune modulation in combination regimens is below currently used levels, supporting dose de-escalation as a strategy to maintain efficacy while improving tolerability.

The QSP model developed in this study demonstrated promising results in identifying biomarkers and optimizing doses for the camrelizumab and apatinib combination regimen. However, it has certain limitations. Firstly, this study used tumor size dynamics in aHCC patients as the core efficacy output, without further evaluation of progression-free or overall survival. Moreover, HBV/HCV status and Child-Pugh classification were not incorporated into the model framework, limiting subgroup analyses. Secondly, we simplified apatinib’s anti-angiogenic effect, which reduces the maximum tumor carrying capacity, into a single parameter (k_K_apat_). While this provides a clearer representation of its inhibitory effect on tumor cell proliferation, it reduces mechanistic interpretability. Future studies will incorporate detailed signaling pathways to better evaluate efficacy, adverse effects, and potential resistance mechanisms (59). Thirdly, due to limited computational resources and the lack of systematically integrated spatial multi-omics data for calibration and validation, intratumoral spatial heterogeneity was not incorporated. Future improvements could leverage single-cell sequencing and spatial transcriptomics to better capture the complexity of the tumor microenvironment. Emerging evidence indicates that, compared with the tumor core, macrophage recruitment and M2 polarization are more pronounced at the tumor margin, leading to localized immunosuppression and accelerated progression of hepatocellular carcinoma (60). These spatially distinct immune features suggest that future QSP models for advanced HCC may benefit from explicitly separating the tumor into core and margin compartments, thereby better capturing the invasive behavior and therapeutic resistance observed in clinical practice. Finally, during the VP generation process, we can utilize the machine learning surrogate modeling approach proposed by Myers et al. to pre-screen parameter combinations while maintaining full model validation, significantly improving the efficiency of VP generation (61).

## 5 Conclusions

This study expands the functionality of the QSP-IO platform to make it applicable to patients with aHCC receiving combination therapy with camrelizumab and apatinib. The model was calibrated and validated using real-world clinical trial data. A virtual clinical trial was conducted based on the established QSP model to simulate the therapeutic outcomes of camrelizumab and apatinib administration. For biomarker identification, we combined subgroup analysis with machine learning approaches, and identified the CD8+/Treg ratio, CD4+/Treg ratio, and the density of MDSCs as potential predictive biomarkers for treatment efficacy. Additionally, the feasibility of reducing the apatinib dose from 250 mg to 125 mg was evaluated, providing a reference for future dose optimization in clinical practice. Overall, this study highlights the significant potential of QSP in biomarker discovery and optimization of therapeutic strategies.

## Data Availability

The original contributions presented in the study are included in the article/**Supplementary Material**. Further inquiries can be directed to the corresponding authors.
